# CaMKII-dependent responses to ischemia and reperfusion challenges in the heart

**DOI:** 10.3389/fphar.2014.00096

**Published:** 2014-05-06

**Authors:** James R. Bell, Martin Vila-Petroff, Lea M. D. Delbridge

**Affiliations:** ^1^Department of Physiology, University of MelbourneMelbourne, VIC, Australia; ^2^Centro de Investigaciones Cardiovasculares, Centro Científico Tecnológico La Plata, Facultad de Ciencias Médicas, Universidad Nacional de La PlataLa Plata, Argentina

**Keywords:** CaMKII, ischemia, reperfusion, contractile function, Ca^2+^ handling, cardiomyocyte death

## Abstract

Ischemic heart disease is a leading cause of death, and there is considerable imperative to identify effective therapeutic interventions. Cardiomyocyte Ca^2+^ overload is a major cause of ischemia and reperfusion injury, initiating a cascade of events culminating in cardiomyocyte death, myocardial dysfunction, and occurrence of lethal arrhythmias. Responsive to fluctuations in intracellular Ca^2+^, Ca^2+^/calmodulin-dependent protein kinase II (CaMKII) has emerged as an enticing therapeutic target in the management of ischemic heart injury. CaMKII is activated early in ischemia and to a greater extent in the first few minutes of reperfusion, at a time when reperfusion arrhythmias are particularly prominent. CaMKII phosphorylates and upregulates many of the key proteins involved in intracellular Na^+^ and Ca^2+^ loading in ischemia and reperfusion. Experimentally, selective inhibition of CaMKII activity reduces cardiomyocyte death and arrhythmic incidence post-ischemia. New evidence is emerging that CaMKII actions in ischemia and reperfusion involve specific splice variant targeted actions, selective and localized post-translational modifications, and organelle-directed substrate interactions. A more complete mechanistic understanding of CaMKII mode of action in ischemia and reperfusion is required to optimize intervention opportunities. This review summarizes the current experimentally derived understanding of CaMKII participation in mediating the pathophysiology of the heart in ischemia and in reperfusion, and highlights priority future research directions.

## ISCHEMIA, REPERFUSION, AND Ca^**2+**^ OVERLOAD

Ischemic heart disease is a leading cause of mortality worldwide. Though considerable advances have been made in the understanding of the processes involved in the pathological consequences of an ischemic event, effective therapeutic management of this disease has been difficult to achieve. Ca^2+^/calmodulin-dependent protein kinase II (CaMKII) has emerged as an enticing therapeutic target in the management of ischemic heart injury, with considerable focus on its inhibition as a means for reducing injury arising from ischemia and reperfusion.

**FIGURE 1:**
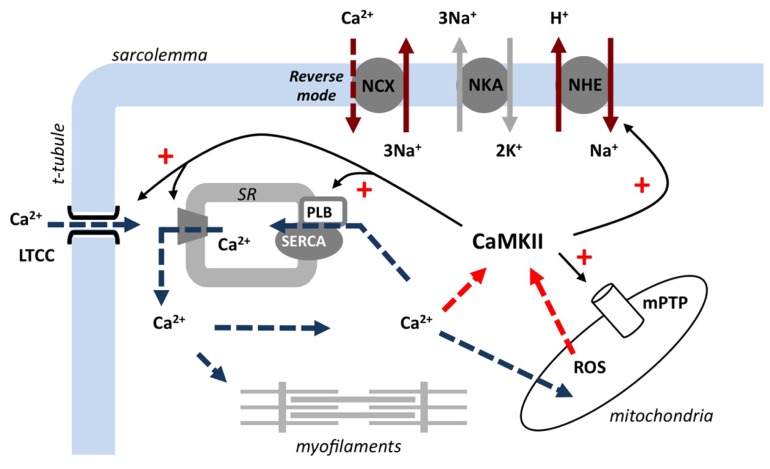
**Overview of CaMKII activation and substrate interaction in reperfusion.** Post-ischemic restoration of coronary flow re-establishes the trans-sarcolemmal proton gradient and stimulates Na^+^/H^+^ exchange. This promotes reverse-mode Na^+^/Ca^2+^ exchange and leads to intracellular Ca^2+^ overload. Ca^2+^-activated CaMKII upregulates many Ca^2+^-related channels/transporters, further increasing cytosolic/mitochondrial Ca^2+^ levels, and triggers the opening of the mitochondrial permeability transition pore. The increase in Ca^2+^ and reactive oxygen species generation creates a positive feedback on CaMKII and exacerbates ischemia/reperfusion injury. NCX, Na^+^/Ca^2+^ exchanger; NHE, Na^+^/H^+^ exchanger; NKA, Na^+^/K^+^-ATPase; LTCC, L-type Ca^2+^ channel; SR, sarcoplasmic reticulum; SERCA2a, sarcoplasmic/endoplasmic reticulum Ca^2+^-ATPase; PLB, phospholamban; ROS, reactive oxygen species; mPTP, mitochondrial permeability transition pore.

A complex series of cardiomyocyte events occur during both ischemia and reperfusion to culminate in an intracellular milieu primed for activating CaMKII. Briefly, insufficient tissue perfusion and oxygen availability in ischemia induce a shift to increased reliance on anaerobic glycolysis for ATP generation, promoting a build-up of metabolic intermediates including lactate and protons. This ionic shift stimulates the Na^+^/H^+^ exchanger (NHE) to maintain physiological pH, which contributes to an accumulation of intracellular Na^+^. The rise in intracellular Na^+^ is exacerbated by a decrease in Na efflux, including a reduction of Na^+^/K^+^-ATPase (NKA) activity that may be related to lower ATP availability (van Echteld et al., 1991). Elevated cytosolic Na^+^ levels reduce the capacity for Ca^2+^ efflux via the Na^+^/Ca^2+^ exchanger (NCX) and promote the Ca^2+^ influx “reverse” operating mode of this transporter. Re-establishing coronary flow (reperfusion) is essential to ultimately allow any salvage of the ischemic myocardium, but involves a significant acute cardiomyocyte jeopardy. Reperfusion rapidly restores the trans-sarcolemmal proton gradient, exacerbating cellular Na^+^ accumulation (concomitant with restoration of NKA activity) and eventually culminating in Ca^2+^ overload. High cytosolic Ca^2+^ levels can have profound negative effects on the cardiomyocyte in reperfusion (Murphy and Steenbergen, 2008), inducing hypercontracture, electrical instability, and contractile dysfunction. Ca^2+^ overload is a major activator of the mitochondrial permeability transition pore (mPTP) in reperfusion, a response associated with reactive oxygen species (ROS) generation and the initiation of pro-death pathways (Halestrap, 2009). As CaMKII is highly sensitive to cytosolic Ca^2+^ levels and regulates many channels and transporters implicated in the steps leading to Ca^2+^ overload, there is considerable scope for a CaMKII-mediated amplification of high Ca^2+^ related pathologies (see **Figure 1**). Furthermore, reports that CaMKII is susceptible to oxidation (ox-CaMKII(Met281/2)), promoting autonomous activation (Erickson et al., 2008; Palomeque et al., 2009), suggests the sub-cellular environment in ischemia (acidosis, high cellular Ca^2+^) and reperfusion (Ca^2+^ overload, oxidative stress) provides an optimal setting for rapid CaMKII activation – and highlights the potential for intervention strategies targeting CaMKII.

## CaMKII IS RAPIDLY ACTIVATED IN ISCHEMIA AND REPERFUSION

Seminal studies involving the characterization of CaMKII activation in *ex vivo* rodent hearts have mapped phospholamban (PLB) phosphorylation at the CaMKII-specific site (P-PLB(Thr17)) as a marker of CaMKII activation during acute ischemia and reperfusion (Vittone et al., 2002). PLB is a regulatory accessory protein to the sarcoplasmic reticulum (SR) Ca-ATPase (SERCA2a), which exerts an inhibitory action on SERCA2a. PLB inhibition is relieved by phosphorylation either by CaMKII or protein kinase A. Numerous subsequent studies, including our own, have shown P-PLB(Thr17) to be briefly elevated in early ischemia, peaking in the initial 1–3 min of reperfusion (Vittone et al., 2002; Said et al., 2003; Valverde et al., 2006; Vila-Petroff et al., 2007; Salas et al., 2010) at a time when hypercontracture and ventricular arrhythmias are prevalent, before rapidly returning to basal activation level. This elevated P-PLB(Thr17) occurs concomitantly with phosphorylation of other CaMKII substrates, including the SR Ca^2+^ release channel (RyR2) and titin (Said et al., 2011; Hidalgo et al., 2013), and can be blocked with CaMKII inhibitors, including KN93 and AIP (Vittone et al., 2002; Said et al., 2003; Valverde et al., 2006; Salas et al., 2010; Hidalgo et al., 2013). The source of Ca^2+^ stimulating CaMKII activity differs in ischemia and reperfusion, with Ca^2+^ entry through the L-type Ca^2+^ channel (LTCC) activating CaMKII in ischemia and the NCX in reperfusion (blocked by nifedipine and KB-R7943 respectively; Vittone et al., 2002). There is evidence that CaMKII is activated both by phasic and tonic shifts in cardiomyocyte intracellular free Ca^2+^ levels, and that local and global Ca^2+^ signals have distinct effects (Wu et al., 2006). The relative importance of each type of stimulus, and the additional influence of post-translational modifications (e.g., oxidation of the regulatory domain at Met281/282) in determining overall CaMKII activation status in ischemia and reperfusion is not fully elucidated.

CaMKII is also undoubtedly influenced by the intracellular acidosis prevalent in ischemia and early reperfusion. Both CaMKII autophosphorylation (P-CaMKII(Thr287)) and P-PLB(Thr17) rapidly increase in acidic conditions, contributing to the recovery of Ca^2+^ transients and contractile function that are initially suppressed in acidic conditions (DeSantiago et al., 2004; Mundina-Weilenmann et al., 2005; Vila-Petroff et al., 2010). Inhibiting CaMKII suppresses PLB phosphorylation (and hence SERCA activity), reducing SR Ca^2+^ uptake, causing cytsolic Ca^2+^ levels to increase (affecting both systolic and diastolic function; see (Mattiazzi and Kranias, 2014)). An increase in P-CaMKII(Thr287) has also been shown in the initial minutes of reperfusion (Said et al., 2011), consistent with an elevated autonomous CaMKII activity recently reported in *in vivo* mouse hearts subjected to 1 h ischemia and 3 min reperfusion (Ling et al., 2013). Interestingly, in contrast to the *ex vivo* studies, this elevated activity *in vivo* was associated with a maintained increase in P-PLB(Thr17) and RyR2 phosphorylation (P-RyR2(Ser2814)) throughout 120 min of subsequent reperfusion. The substantial activation of CaMKII, which occurs in ischemia and reperfusion, would be expected to be an important determinant of cardiomyocyte Ca^2+^ homeostasis and post-ischemic outcomes.

## CaMKII EXACERBATES ISCHEMIC INJURY

**FIGURE 2:**
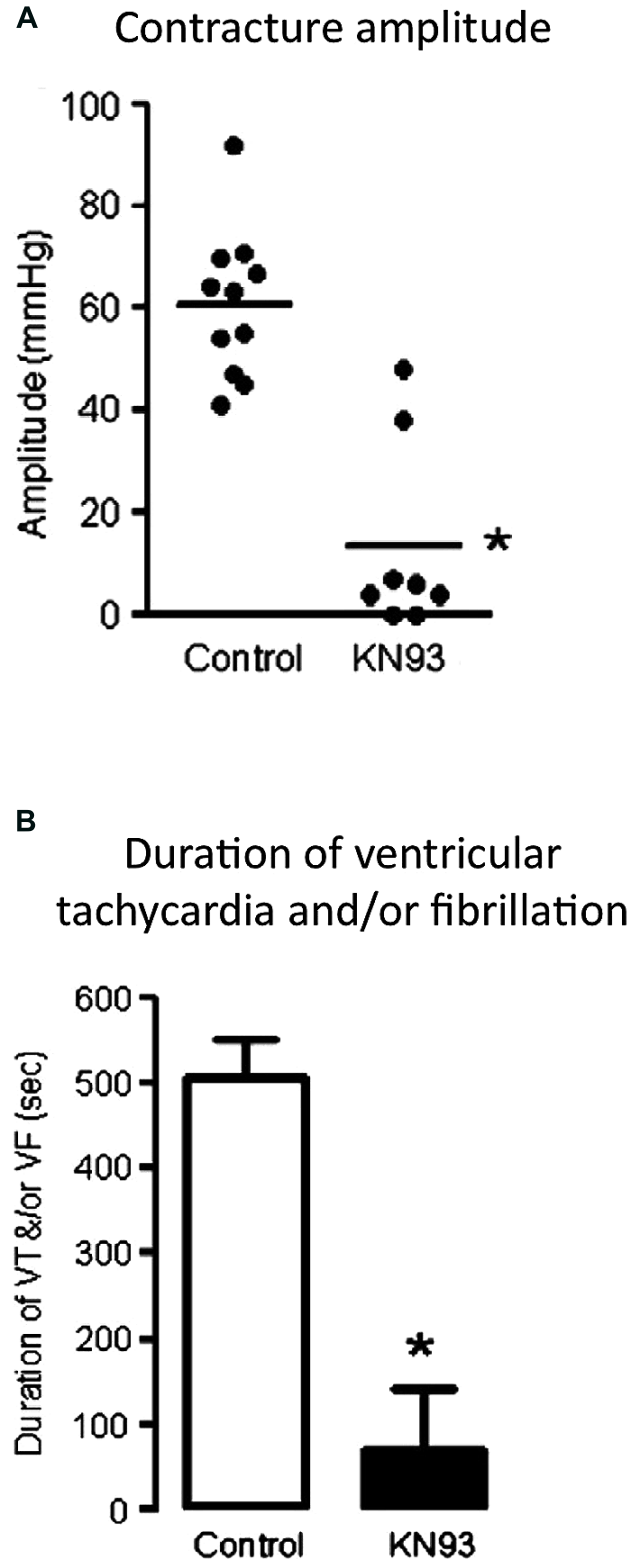
**CaMKII inhibition reduces ischemic contracture and reperfusion arrhythmias in male ex vivo hearts.** Hearts subjected to ischemia/reperfusion were treated with a CaMKII inhibitor (KN93). **(A)** The amplitude of ischemic contracture was significantly lower in the presence of KN93, indicating CaMKII contributes to cardiomyocyte Ca^2+^ loading in ischemia. **(B)** Analysis of ventricular pressure traces showed a substantial reduction in the total duration of ventricular tachycardia and/or fibrillation in the first ten minutes of reperfusion in KN93-treated hearts. Data are expressed as mean ± SEM, *p <0.05 vs. control. Reproduced with permission (Elsevier, (Bell et al., 2012)).

Increased intracellular Ca^2+^ correlates with the onset of irreversible injury in ischemia (Murphy and Steenbergen, 2008). Inhibiting Ca entry through the LTCC has been shown to prevent/delay ischemic contracture onset and arrhythmias (Henry et al., 1977; Curtis et al., 1984; Curtis and Walker, 1988). Considering CaMKII activation in early ischemia is linked with Ca entry through the LTCC (Vittone et al., 2002), it may be predicted that CaMKII contributes to the cascade of events leading to ischemic pathogenesis. We have shown that inhibiting CaMKII with KN93 significantly delays and blunts the extent of ischemic contracture in *ex vivo* hearts subjected to 20 min of global ischemia (**Figure 2**, (Bell et al., 2012)), suggesting a role for CaMKII in ischemic myocyte Ca^2+^ loading and the onset of irreversible injury. Inhibiting CaMKII also protects the heart in a chronic *in vivo* ischemic setting, as demonstrated in studies utilizing two different CaMKII inhibitor rodent expression models (CaMKII inhibitor peptide, AC3-I; mitochondrial-specific CaMKII inhibitor protein, mtCaMKIIN). These studies found that CaMKII promotes apoptosis *in vivo* (5 h post-myocardial infarction) by exacerbating SR and/or mitochondrial Ca^2+^ loading (Yang et al., 2006; Joiner et al., 2012).

## CaMKII ACTIONS IN REPERFUSION RECOVERY ARE DEPENDENT ON ISCHEMIC DURATION

**FIGURE 3:**
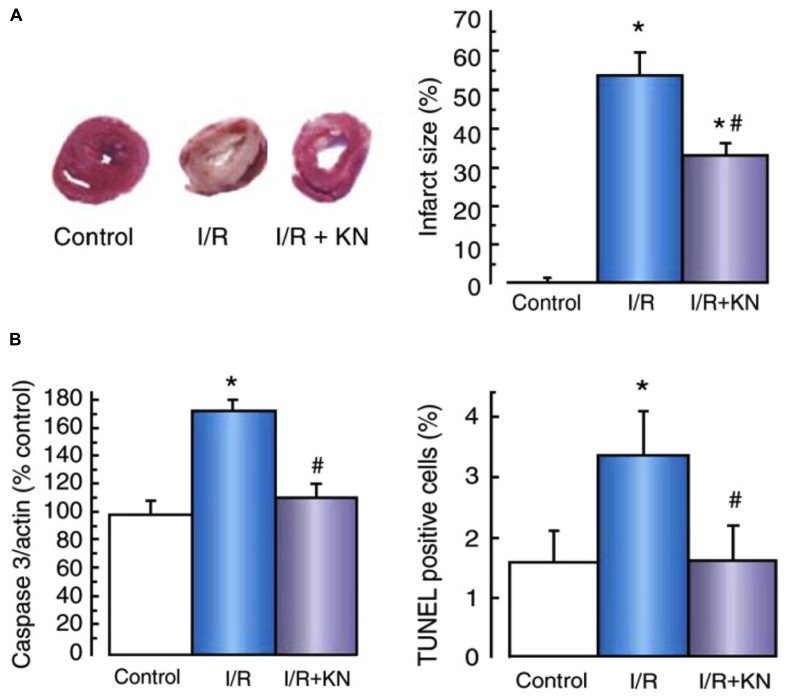
**Cell death in ischemia/reperfusion was suppressed by CaMKII inhibition. (A)** Assessment of TTC stained heart cross-sections showed KN93 significantly reduced infarct size in ischemia/reperfusion. **(B)** Lower caspase 3 activation and less TUNEL positive cells in these hearts showed KN93 also reduced apoptosis in reperfusion. Data are expressed as mean ± SEM, *p <0.05 vs. control; ^#^*p* <0.05 vs. I/R. Reproduced with permission (Oxford Journals, (Vila-Petroff et al., 2007)).

While initial studies assessing CaMKII in post-ischemic reperfusion suggested that CaMKII activation may improve functional recovery, subsequent studies have reported different findings and suggest that the role played by CaMKII in the dysfunction associated with reperfusion may be dependent on the duration of the preceding ischemic insult. In isolated hearts subjected to 20 min of ischemia, recovery of left ventricular developed pressure in reperfusion was lower and diastolic dysfunction exacerbated in the presence of KN93 (Vittone et al., 2002; Said et al., 2003). This poor recovery was attributed to a disruption of CaMKII actions on SR Ca^2+^ uptake, as P-PLB(Thr17) was suppressed in early reperfusion. Parallel experiments were conducted on hearts from mutant mice expressing a PLB Thr to Ala mutation at amino acid residue 17 (Said et al., 2003), such that CaMKII-mediated phosphorylation at this site could not occur. The inhibitory action of PLB on SERCA cannot be relieved in these mice. These hearts exhibited a comparable contractile dysfunction in reperfusion as was observed in wild-type hearts treated with KN93. Taken together, these observations indicate that CaMKII can be beneficial in reperfusion, augmenting SR Ca^2+^ uptake in reperfusion and enhancing cytosolic Ca^2+^ clearance. However, with a longer ischemic challenge, these benefits attributed to CaMKII activation in modulating reperfusion response are lost. Our further studies showed that extending the duration of ischemia from 20 to 45 min in *ex vivo* hearts profoundly affects how CaMKII influences reperfusion outcomes. Indeed, treating hearts with KN93 significantly reduces infarct size and apoptosis, and improves functional recovery (**Figure 3**, Vila-Petroff et al., 2007). Isolated cardiomyocyte survival is improved in simulated ischemia and reperfusion with KN93 and AIP, to an extent equivalent to the improvement achieved by inhibition either of reverse-mode NCX or SR Ca^2+^ cycling (SERCA2a and/or RyR2; (Vila-Petroff et al., 2007; Salas et al., 2010)). These data suggests that following shorter durations of ischemia, CaMKII activation augments SERCA2a activity to enhance cytosolic Ca^2+^ removal and reduce reperfusion injury/dysfunction. However, with more extensive ischemia, the influence of CaMKII on SR Ca^2+^ release mechanisms become more prominent in reperfusion, such that SR Ca^2+^ leak exceeds the capacity of SERCA2a to reuptake cytosolic Ca^2+^, and Ca^2+^-triggered reperfusion pathologies are exacerbated.

Phosphorylation of the RyR2 (P-RyR2(Ser2814)) by CaMKII increases the open probability of the SR Ca^2+^ release channel, augmenting SR Ca^2+^ leak and elevating cytosolic Ca^2+^ levels (Wehrens, 2011). A CaMKII-mediated increase in SR Ca^2+^ release channel leakiness with elevation of cytosolic Ca^2+^ levels has important implications for mitochondrial function and myocyte viability. Studies indicate that inhibiting CaMKII with KN93 may lower SR Ca^2+^ load to suppress cytochrome C release and mitochondrial swelling in reperfusion (Salas et al., 2010), both of which activate pro-death pathways in the heart. While this mitochondrial response partly reflects the close beat-to-beat relationship between cytosolic and mitochondrial Ca^2+^ levels (Robert et al., 2001), there is also evidence that CaMKII directly influences mitochondrial operation in ischemia/reperfusion (Joiner et al., 2012). Hearts from mice overexpressing a highly specific CaMKII inhibitor localized to the mitochondria (mtCaMKIIN) exhibit less mitochondrial injury and apoptosis, smaller infarcts, and recover contractile function to a greater degree (vs. wild-type). Evidence suggests the actions of CaMKII in the mitochondria are multi-faceted, including a direct phosphorylation of the mitochondrial Ca^2+^ uniporter to increase the uniporter current (Joiner et al., 2012). This would increase mitochondrial Ca levels, leading to mPTP opening and dissipation of the mitochondrial inner membrane potential. Furthermore, a resultant increase in ROS production would be expected to exacerbate CaMKII activation and actions.

Beyond these mitochondrial actions, recent data suggests that the role of CaMKII in exacerbating ischemia and reperfusion pathologies is not restricted to disrupting Ca^2+^ homeostasis at the SR and mitochondria, and may extend to direct pro-inflammatory actions. Indeed, cardiac-specific deletion of CaMKIIδ reduces chronic post-ischemic reperfusion (24 h) inflammation and injury *in vivo*, diminishing IκBα degradation and NF-κB activation (Ling et al., 2013). NF-κB is well recognized as an important pro-inflammatory mediator in ischemia and reperfusion (Van der Heiden et al., 2010), and this more recent evidence of a direct stimulatory action on NF-κB and the inflammatory response extends understanding of CaMKII as a regulator of the ischemic myocardial stress response.

## CaMKII MODULATION OF RyR2 DETERMINES REPERFUSION ARRHYTHMOGENESIS

CaMKII has been implicated in generating arrhythmias in numerous different cardiopathologies (Rokita and Anderson, 2012), primarily attributed to its stimulatory action on RyR2. Augmented P-RyR2(Ser2814) increases SR Ca^2+^ leak and promotes Ca^2+^ extrusion though Na^+^/Ca^2+^ exchange. This electrogenic process partially depolarizes the cell, increasing the likelihood of spontaneous contraction. Experimentally, inhibiting CaMKII reduces spontaneous beats in post-acidotic and pro-oxidant environments (Said et al., 2008; Xie et al., 2009), both of which play a central role in the pathogenesis of ischemia and reperfusion injury. We have shown that CaMKII inhibition with KN93 suppresses the incidence of lethal arrhythmias (ventricular tachycardia and/or fibrillation) in early reperfusion through an unknown mechanism (**Figure 2**, (Bell et al., 2012)). There are reservations regarding the use of KN93 as a CaMKII inhibitor, due to its reported non-selective actions on other transporters including the L-type Ca and potassium channels. The occurrence of these non-selective actions depends on treatment duration and dose (Gao et al., 2006; Rezazadeh et al., 2006). Studies utilizing a similar isolated heart preparation and CaMKII inhibition protocol also report an antiarrhythmic action with KN93, that is absent in parallel studies using the inactive analog, KN92 (Said et al., 2011). This suggests a legitimate role for CaMKII in promoting arrhythmias in reperfusion. Indeed, these studies showed that inhibiting CaMKII reduces incidence of ventricular premature beats, primarily through a suppression of CaMKII mediated SR Ca release channel phosphorylation (at RyR2(Ser2814)) and associated SR Ca leak (Said et al., 2011). CaMKII-dependent phosphorylation of the SR Ca channel increases channel open probability, lowering the SR Ca threshold and increasing the propensity for Ca waves (Curran et al., 2010; Stokke et al., 2011). This ability to modulate the SR Ca channel and its influence on self-propagating SR Ca release indicates that inhibiting CaMKII at the time of reperfusion may have therapeutic potential as a first-line antiarrhythmic agent.

It should be noted that CaMKII actions are dependent on the balance of substrate phoshorylation by CaMKII and dephosphorylation by associated protein phosphatases (PPs) in the myocyte, including PP1 and PP2a. Downstream Ca handling protein targets of CaMKII are regulated by these phosphatases. Indeed, PP1 is reported to form part of a multimeric complex in the regulation of PLB (as reviewed previously, (Mattiazzi and Kranias, 2014)), which may influence CaMKII-mediated phosphorylation, cardiac contractility and the ischemic stress response (Nicolaou et al., 2009). The balance between CaMKII and phosphatase activation will therefore clearly influence the relative actions of CaMKII in the heart. Furthermore, CaMKII autophosphorylation is itself regulated by phosphatases including PP1, PP2a, and CaMK phosphatase (CaMKP; Ishida et al., 2008), though little is known about how these regulate myocardial CaMKII. Very recently, it has been shown that the CaMKP is expressed in the heart, and changes in response to systemic loading (Previlon et al., 2014). Interestingly, basal CaMKP levels were higher in females (vs. males) and loading-induced CaMKP expressions were sex-specific. It is important to highlight that all the CaMKII studies discussed above have involved experimental studies of male rodent cardiac tissues/myocytes only. There are well-described sex differences in excitation–contraction coupling and cardiomyocyte Ca^2+^ handling processes, and the myocardial response to ischemia and reperfusion (Bell et al., 2013a). Limited experimental evidence indicates that CaMKII activity is influenced by sex and sex steroids (Konhilas et al., 2004), and that estrogen may suppress CaMKII actions in ischemia and reperfusion (Ma et al., 2009). However, our very recent data show that P-CaMKII(Thr287) and P-PLB(Thr17) levels are augmented in reperfused female hearts (vs. male controls), despite these hearts exhibiting fewer arrhythmias in reperfusion (Bell et al., 2013b). These findings suggest that CaMKII is not always pro-arrhythmic in reperfusion, and support the concept that CaMKII can have both beneficial and detrimental actions in reperfusion which may depend on the sub-cellular environment in which the enzyme is activated.

## EVIDENCE FOR A BENEFICIAL ROLE FOR CaMKII IN ISCHEMIA AND REPERFUSION

Though considerable evidence indicates a deleterious role for CaMKII in ischemia and reperfusion, a growing body of work suggests there may be settings where CaMKII is beneficial. CaMKII has been implicated in the cardioprotection afforded by ischemic preconditioning (Osada et al., 2000; Benter et al., 2005; Li et al., 2007) and may form part of the signaling cascade that culminates in opening of the end-effector K_ATP_ channels (Li et al., 2007; Chai et al., 2011; Zhang et al., 2014). Other data also suggest that CaMKIIδ splice variants may exert differential responses in the pathological environments prevalent in ischemia and reperfusion. Peng et al. (2010) showed that CaMKIIδ_B_ has been shown to protect against apoptosis in hydrogen peroxide-treated neonatal rat ventricular myocytes, through an upregulation of heat shock protein 70 (Peng et al., 2010). However, expression of CaMKIIδ_B_ decreases in these conditions over a period of hours, in direct contrast to CaMKIIδ_C_, such that a reduction of influence on ischemic resilience would be expected to reduce with time (Peng et al., 2010). Interestingly, preliminary reports indicate that hearts from transgenic mice selectively overexpressing CaMKIIδ_B_ are less susceptible to ischemia and reperfusion injury (Gray et al., 2013), corroborating the notion of a cardioprotective action for this splice variant in ischemia/reperfusion.

As outlined above, CaMKII can provide important inotropic support to maintain contractile function in stunned hearts subjected to a brief ischemic challenge. An important body of experimental work supports the view that CaMKII actions can transition from conferring benefit to liability in a manner which reflects the myocyte capacity to balance relative SR Ca^2+^ uptake and leak. CaMKII “protection” is understood to be forfeited when the greater probability of spontaneous SR Ca^2+^ release outweighs the enhancement of cytosolic Ca^2+^ clearance (Mattiazzi and Kranias, 2011). The mechanisms responsible are not fully understood, but the extent of ox-CaMKII(Met281/2) generation may be implicated. With extended ischemia, it may be that an increase in ROS production in reperfusion further augments CaMKII activation and actions, possibly through greater ox-CaMKII(Met281/2) generation. As ox-CaMKII(Met281/2) increases P-RyR2(Ser2814) phosphorylation (Ho et al., 2014), an increase in SR Ca^2+^ leak would be expected. This may be a critical factor in promoting injury in reperfusion. Our very recent findings suggest that specific post-translational modifications of CaMKII determine substrate specificity, and that in contrast to P-CaMKII(Thr287), ox-CaMKII(Met281/2) selectively phosphorylates the RyR2 and not PLB (Bell et al., 2014). Augmented SR Ca uptake would be predicted to increase SR Ca^2+^ load (Shannon et al., 2002; Venetucci et al., 2007). With a concomitant increase in P-RyR2(Ser2814), this could augment SR Ca^2+^ leak; an effect that may be offset if the Ca^2+^ levels at the RyR2 cytoplasmic domain are sufficiently reduced (Laver, 2009).

In summary, CaMKII is a crucial regulatory intermediate in ischemia and reperfusion injury. CaMKII is relatively quiescent under basal conditions, but displays considerable capacity to exacerbate Ca^2+^ mismanagement and mitochondrial dysfunction in response to ischemic changes in cellular Ca^2+^ and redox status. Thus, there is potential for CaMKII inhibitor utilization as a prophylactic therapeutic intervention for “at risk” patients with ischemic heart disease. Conversely, conflicting reports suggest that in other circumstances maintained CaMKII activation can improve reperfusion recovery. To optimize and tailor therapeutic strategies involving manipulation of CaMKII activation, it is clear that further mechanistic studies are required to fully understand the nuances of CaMKII response in a range of pathophysiologic settings.

## Conflict of Interest Statement

The authors declare that the research was conducted in the absence of any commercial or financial relationships that could be construed as a potential conflict of interest.
